# Public health round-up

**DOI:** 10.2471/BLT.21.010221

**Published:** 2021-02-01

**Hygiene in the pandemic**

A midwife washes her hands at a mother and child centre in Hargeisa, Somalia. Around 1.8 billion people are at heightened risk of COVID-19 and other diseases because they use or work in health-care facilities without basic water services, according to a new global progress report on water, sanitation and hygiene.

**Figure:**
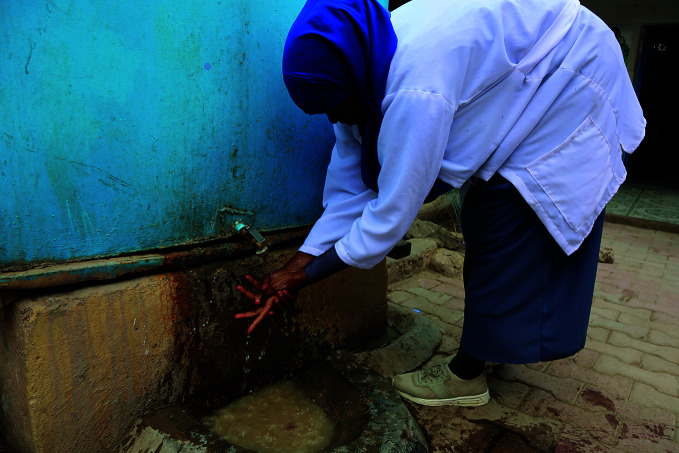
WHO/Ilyas Ahmed

## SARS-CoV-2 variants raise concerns

SARS-CoV-2 variants were reported in Denmark, the United Kingdom of Great Britain and Northern Ireland, and South Africa at the end of 2020, raising concerns about transmissibility, pathogenicity and possible implications for diagnostic tests and vaccines.

One of the mutations in the United Kingdom variant was found to affect the performance of some polymerase chain reaction diagnostic tests. Another mutation, found in both the United Kingdom and South Africa variants, is in the virus’s receptor binding domain. Because several of the vaccines currently being developed or deployed target receptor binding, researchers are seeking to determine if the mutation impacts vaccine performance.

The World Health Organization (WHO) moved to expand its scientific collaboration and monitoring of emerging variants of SARS-CoV-2, and on 12 January convened a day-long virtual meeting which brought together 1750 scientists from around the globe to discuss critical knowledge gaps and research priorities.

WHO recommends that authorities continue to strengthen disease control activities, including the monitoring of their epidemics through surveillance, testing, contact tracing and outbreak investigation, and adjusting public health and social measures to reduce virus transmission, when required. Where feasible, WHO also recommends increased routine systematic sequencing of SARS-CoV-2 viruses to better understand SARS-CoV-2 transmission and to monitor the emergence of variants.

http://bit.ly/38G6wGW

## Sequencing SARS-CoV-2

WHO issued interim guidance on the genomic sequencing of SARS-CoV-2. Released on 8 January, the document provides national-level policy-makers and stakeholders with guidance on how to maximize the public health benefit of SARS-CoV-2 genomic sequencing activities in the short and long term. WHO also released a guide designed to help public health technical officers and laboratories responsible for, or considering the establishment of, SARS-CoV-2 genomic sequencing programmes.

http://bit.ly/2Lp9jvb

http://bit.ly/35wHkjY

## Vaccine approved for emergency use

WHO listed the Pfizer/BioNTech COVID-19 mRNA vaccine BNT162b2 for emergency use, making it the first COVID-19 vaccine to receive emergency validation from WHO.

Regulatory experts determined that the vaccine met the Organization’s safety and efficacy criteria, and that the benefits of using the vaccine outweighed potential risks.

Announced on 31 December 2020, the Emergency Use Listing opens the door for countries wishing to expedite their own regulatory approval processes to import and administer the vaccine. It also enables the United Nations Children’s Fund (UNICEF) and the Pan American Health Organization to procure the vaccine for distribution to countries in need.

WHO and its partners are evaluating other vaccines for emergency listing and encourage vaccine developers to come forward with suitable products for assessment.

http://bit.ly/2Lt5tkL

## COVAX secures new vaccines

COVAX – a global initiative designed to ensure that COVID-19 vaccines are available worldwide to countries of all incomes – has secured access to 670 million doses of promising vaccine candidates from developers and manufacturers.

Announced on 18 December 2020, the deals include an advance purchase agreement with AstraZeneca for 170 million doses of the AstraZeneca/Oxford candidate, and a memorandum of understanding with Johnson & Johnson for 500 million doses of the Janssen candidate vaccine.

COVAX already has deals with the Serum Institute of India for 200 million doses of either the AstraZeneca/Oxford or Novavax candidates with options for up to 900 million doses more, as well as a statement of intent for 200 million doses of the Sanofi/GSK vaccine candidate.

In addition, COVAX has secured first right of refusal deals to potentially more than one billion doses of candidate vaccines in the COVAX research and development portfolio that will be produced if proven safe and efficacious in clinical trials.

http://bit.ly/3bwndX2

## Ebola vaccine stockpile

Four international health and humanitarian organizations announced the establishment of a global Ebola vaccine stockpile to support outbreak response. The vaccine is the injectable single-dose Ebola vaccine (rVSV∆G-ZEBOV-GP, live) which provides protection against the Zaire Ebola virus species, the most common cause of outbreaks.

The stockpile, which is stored in Switzerland, will allow countries, with the support of humanitarian organizations, to contain future Ebola virus disease outbreaks. The targeted overall delivery time from the stockpile to countries is seven days. At 6890 doses, the stockpile is currently well short of the Strategic Advisory Group of Experts-recommended level of 500 000 doses and will be built up over time.

Announced on 11 January, the initiative was led by the International Coordinating Group on Vaccine Provision, which includes WHO, UNICEF, the International Federation of Red Cross and Red Crescent Societies, and Médecins Sans Frontières, with financial support from Gavi, the Vaccine Alliance.

http://bit.ly/2LvJnOm

## WASH and the pandemic

Around 1.8 billion people are at heightened risk of infection with COVID-19 and other diseases because they use or work in health-care facilities without basic water, sanitation and hygiene (WASH) services. This is according to the *Global progress report on WASH in health care facilities: fundamentals first*, which was published by WHO and UNICEF on 14 December and reveals how COVID-19 is exposing key vulnerabilities within health systems, including inadequate infection prevention and control.

http://bit.ly/2LIBLYT

## Refugees and migrants in the pandemic

The COVID-19 pandemic has taken a significant toll on the mental and physical health of refugees and migrants according to a new WHO survey. More than 30 000 refugees and migrants from around the world participated in the survey which was published on 18 December. Over half reported greater levels of depression, fear, anxiety and loneliness as a result of the pandemic, while one in five reported a deterioration of mental health and increased use of drugs and alcohol. The survey underlines the need to include refugees and migrants in policy responses to COVID-19.

http://bit.ly/39tmIdK

## Cholera in Togo

Health authorities in Togo implemented a plan to contain an outbreak of cholera in the city of Lomé. The outbreak was declared by the Minister of Health, Public Hygiene and Universal Access to Care of Togo on 24 November 2020.

From 11 November to 28 December 2020, a total of 67 people with suspected cholera infections had been identified, two of whom had died. Of the 67, 19 were fishermen and as of 4 January the health zones concerned were mostly located in port areas where poor hygiene and sanitation conditions prevail. Two of the infections were reported in the Netherlands in travellers from Togo and there was a possibility of further exported cases.

http://bit.ly/39pMOOE

**Cover photo**

People getting water at a pump in the province of Cabo Delgado, Mozambique, where the population have been suffering as a result of floods just one year after tropical cyclones Kenneth and Idai devastated the region.

**Figure:**
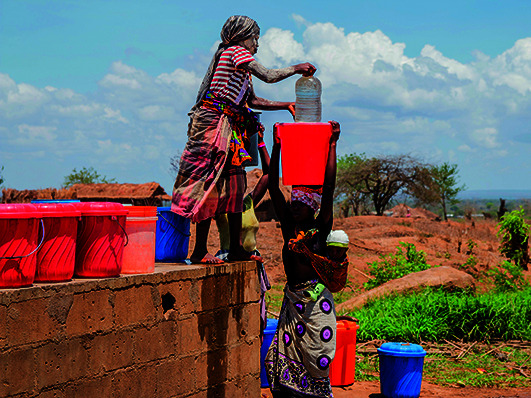
UNICEF/Mauricio Bisol

## Strengthening public food procurement

WHO and partners launched an action framework to support the development and implementation of public food procurement and service policies. Launched on 12 January, the framework includes nutrition criteria for food served and sold in public settings, the intention being to reduce preventable diseases and deaths from excessive consumption of salt, sugars and fats, particularly trans fats, and inadequate consumption of whole grains, legumes, vegetables and fruit.

http://bit.ly/3nEMeSb

## Measuring healthy ageing

Some 142 million people aged 60 years and over are currently unable to meet all their basic daily needs according to the *Baseline report for the Decade of Healthy Ageing*, released by WHO on 17 December.

The report presents data for measuring healthy ageing which is defined by WHO as “the process of developing and maintaining the functional ability that enables well-being in older age.” Optimizing “functional ability” is the goal of the Decade of Healthy Ageing, which begins in 2021 and addresses five interrelated abilities that all older people should enjoy, including the ability to meet basic needs.

http://bit.ly/3skOWjq

## Mobilizing youth against COVID-19

A global initiative to invest in and scale up youth-led solutions and engagements in response to COVID-19 was launched by an alliance of the world’s largest youth movements and organizations, together with WHO and the United Nations Foundation. Drawing on the intelligence and enthusiasm of 250 million young people, the initiative aims to support the design and implementation of pandemic response efforts.

http://bit.ly/38Ez977

**Looking ahead**

22–25 February: Fourth Global Vaccine and Immunization Research Forum. http://bit.ly/2Lq16qs

3 March: World Hearing Day 2021. http://bit.ly/3nJ39Tz

